# Integrating quantum synchronization in future generation networks

**DOI:** 10.1038/s41598-025-92038-0

**Published:** 2025-03-04

**Authors:** Swaraj Shekhar Nande, Muhammad Idham Habibie, Milad Ghadimi, Andrea Garbugli, Koteswararao Kondepu, Riccardo Bassoli, Frank H. P. Fitzek

**Affiliations:** 1 Deutsche Telekom Chair of Communication Networks, Technische Universität Dresden, 01069 Dresden, Germany; 2 Cluster of Excellence, Centre for Tactile Internet with Human-in-the-Loop (CeTI), 01069 Dresden, Germany; 3 Department of Computer Science and Engineering, IIT Dharwad, Dharwad, 580007 India; 4 Department of Computer Science and Engineering, University of Bologna, 40126 Bologna, Italy

**Keywords:** Optics and photonics, Physics, Electrical and electronic engineering

## Abstract

The advent of Beyond 5G (emerging 6G) technologies represents a significant step forward in telecommunications, offering unprecedented data speeds and connectivity. These advances enable a wide range of applications, from enhanced mobile broadband and the Internet of Things to ultra-reliable low-latency communication and the tactical Internet. Thus, having accurate and dependable time synchronization is of utmost importance and plays a critical role in ensuring that all processes function smoothly and effectively. However, existing standards, such as the precision time protocol, are unreliable due to jitters, datagram losses, and complexity. Increasing the synchronization error from the ideal tens of nanoseconds to hundreds of microseconds is unacceptable in future-generation networks. This work provides a novel way to establish ultraprecise synchronization, which is critical for the growth of converged optical communication networks and the 6G era. We investigate quantum non-linear synchronization (QNS), which explores the interaction between the non-linear dynamics of atomic systems and dissipation to establish a stable limit-cycle state. In this process, atoms confined within optical resonators are subjected to potential fields, and their spatial motion is synchronized by achieving a stable, phase-locked configuration. By introducing photons into the optical resonators and precisely managing the dissipation effects, it is possible to synchronize multiple optical resonators (referred to as nodes), even in systems with more than three interconnected resonators containing non-linear atoms. To transcend the synchronization signal from the optical setup to communication networks, we propose a distinct mechanism that utilizes the exceptional precision of QNS in the optical lattice setup and frequency down-conversion using frequency combs. In addition, it is combined with electronic components such as analog-to-digital converters and field-programmable gate arrays (FPGAs) to create synchronized digital signals that are understandable to communication networks. Our method transforms optical pulses into precisely timed electrical signals that can be analyzed and used in sophisticated network systems. We demonstrated that QNS and dissipation can synchronize a tri-node clock network to the highest precision of thulium atom-based optical lattice clocks. Our work also highlights the practicality of these applications through MATLAB simulations, bridging theoretical principles and real-world solutions with current technology. In our simulations, we utilized an optical signal with a frequency of 263 THz, downconverted to a lower microwave frequency of 100 GHz to achieve subnanosecond-level synchronized signals. The down-converted signal was subjected to white noise and subsequently digitized. The digital signal was then simulated by sampling rate of $f_s = 100$ GHz or GSa/s (gigasample per second) and limiting the resolution to $b = 8$ bits. Finally, high-frequency noise was removed by implementing low-pass filtration using FPGAs. This study takes an essential step toward meeting the rising demands for rapid and efficient data transfer in the ever-evolving digital communications landscape, enabling faster and more reliable connectivity for future communication networks and the quantum Internet.

## Introduction

The transition from 5G to 6G technology marks a significant leap in telecommunications, bringing faster data speeds and more reliable connections. This advancement will revolutionize applications like high-speed internet, smart devices in the Internet of Things (IoT), and communication systems requiring ultra-low delays. Central to these innovations is precise time synchronization, which ensures seamless interaction in our connected digital world. Synchronization in telecommunications, particularly in the context of URLLC and Tactile Internet, ensures continuous, real-time interaction between multiple devices and systems, a prerequisite for applications such as autonomous driving, telemedicine, and industrial automation.

Current state-of-the-art synchronization technologies exploit packet-based networks, and protocols such as network time protocol (NTP), precision time protocol (PTP), and synchronous ethernet (syncE) are prominent representatives. However, as the development of 5G advances, the limitations of current solutions such as precision time protocol (PTP) become increasingly apparent. The use cases are becoming more relevant; let us mention the requirement of uplink time difference of arrival (UL-TDoA)^1,2^, which requires high precision to locate devices over the 5G network with a centimeter-level accuracy. For example, the strict system requirement assumes 1 ns accuracy of clock synchronization with localization accuracy of less than 50 cm^1^. In particular, experimental evaluations of the White Rabbit (WR) protocol in cascade-chain configurations demonstrated synchronization skew values of $-212.51 \, \text {ps} \pm 45.653 \, \text {ps}$, $-500.66 \, \text {ps} \pm 174.50 \, \text {ps}$, and $-573.45 \, \text {ps} \pm 490.17 \, \text {ps}$ for the 10th, 15th, and 18th nodes, respectively, with a peak-to-peak value of 2.6487 ns at the 18th node^3^. Additionally, we can foresee the next requirement of 6G; the envision enforces a nanosecond level synchronization, which can be applied widely with other applications. Thus, this requires an accurate timing propagation in the network, probably less than $\le 1$ ns^4^. Classical protocols have achieved some level of synchronization on the order of $<100$-picoseconds under optimal conditions but have scalability and energy issues, e.g. white rabbit precision time protocol (PTP-WR)^5^.

We investigated *quantum nonlinear synchronization (QNS)* by exploiting the non-linear atom-field dynamics in a cavity-enhanced optical lattice containing thulium atoms at a magic wavelength of $814.5\,\textrm{nm}$, which provides a clock transition at $= 1.14\,\upmu \textrm{m}$ ($= 263\,\textrm{THz}$ in frequency terms)^6^. By fine-tuning dissipation, a limit-cycle state is generated, synchronizing multiple optical lattice clocks^7^. This process can be stabilized with photonic pumping and mode-locked lasers (MLLs), widely used to generate optical frequency combs essential for high-accuracy synchronization^8^. Recent advancements in MLLs have achieved broad bandwidths, such as the $30\,\textrm{GHz}$ electro-optic frequency comb. Spanning $300\,\textrm{THz}$ in the near-infrared and visible spectrum, as reported by Metcalf et al.^9^. These developments significantly enhance the potential of MLLs for ultrafast precision timing applications. Thus, we foresee MLLs generating frequency combs that can transcend the precision from a $263\,\textrm{THz}$ optical signal to $100\,\textrm{GHz}$. Particulary, the W band of the microwave portion of the electromagnetic spectrum spans 75 to 110 GHz (wavelengths of 2.7–4 mm). It is situated above the IEEE-designated V band (40–75 GHz) and overlaps with NATO’s M band (60–100 GHz).

The next point to consider in this process is to convert these electromagnetic signals into discrete electrical signals that are generally performed by state-of-the-art analog-to-digital converters (ADCs), which can achieve sampling rates of up to 10 GSa/s (gigasamples per second), some approaching 100 GSa/s in specialized applications^10^. Field-programmable gate arrays (FPGAs) can interface with these high-speed ADCs to process digitized signals in real-time, utilizing techniques such as time interleaving and SERDES (serializer/deserializer) blocks to handle the high data rates^11^. After digitization, FPGA-based processing minimizes noise, enhances synchronization, and ensures phase alignment, achieving mean squared errors around $10^{-5}$ and Allan deviations near $10^{-15}$. Current high-performance FPGAs can process data streams at $100\,\textrm{GHz}$^12^. However, further advancements in integrated photonics and ADCs technology are crucial for achieving scalable femtosecond-level synchronization across large networks. By integrating QNS, broadband mode-locked lasers (MLLs), and high-speed frequency combs, our approach lays a robust foundation for femtosecond-level synchronization, addressing the demands of 6G networks, quantum communication, and global timekeeping infrastructures. Current technologies are adequate for laboratory demonstrations, but ongoing innovation is essential to fully exploit the sub-picosecond capabilities of advanced optical clock systems^13^.

To enhance synchronization precision in large-scale communication networks, critical electronic components such as ADCs and field-programmable gate arrays (FPGAs) are employed. The extraordinary accuracy of optical lattice clocks, governed by precise optical transitions, is vital for maintaining data synchronization across expansive and complex network infrastructures^14,15^. In addition, alternative quantum-based synchronization methods have been explored. For instance, researchers^16,17^ have proposed approaches based on photon correlations. These methods utilize intrinsic quantum properties, such as entanglement, to achieve synchronization. By synchronizing correlated photons in space, sub-nanosecond-level synchronization can be realized, offering a complementary pathway for achieving ultra-precise timing and synchronization^17^.

In our work, we utilize the QNS system, which employs photon pumping and their dissipation into the environment for clock synchronization, achieving a precision of $2.62975 \times 10^{14}$ signals per second for a network of clocks^7^. This precision, combined with frequency combs, enables precise timing of optical signals in communication networks, meeting the stringent requirements of future networks^18^. Advanced communication technologies and network architectures like these^7,19^ can improve global time standards. Our research, supported by MATLAB simulations, demonstrates that our synchronization method performs effectively under conditions resembling real-world networks. We achieved femtosecond-level synchronization using a 263 THz optical lattice clock signal downconverted to a microwave frequency of 100 GHz via frequency combs. Key results include a low Allan deviation of $5.62 \times 10^{-15}$, indicating exceptional frequency stability, and a minimum mean squared error (MMSE) $= 2.6 \times 10^{-5}$ between the downconverted and FPGA-filtered signals, reflecting strong synchronization accuracy. Future work will focus on enhancing noise resilience in FPGA-based filtering, extending synchronization to larger networks, and addressing environmental factors affecting the precision of optical lattice clocks.

This work explores synchronization methodologies that integrate emerging technologies operating at the cutting edge of current experimental research. The technologies employed in this study are not yet widely adopted in standard applications. Instead, we focus on their potential rather than their current prevalence. The goal is to demonstrate the feasibility of these technologies and inspire the development of new, large-scale implementations that could shape the future of synchronization and communication systems.

The following sections are structured: section “Literature review” presents a comprehensive literature review outlining existing research in the domain. Section “Quantum synchronization over the network” delves into quantum synchronization within networks, discussing system components, theoretical underpinnings, and spectral density considerations. Section “Optical lattice clocks” focuses on optical lattice clocks, detailing their advantages, femtosecond-level synchronization capabilities, and pulse generation methods. Section “Frequency combs” analyzes frequency combs, emphasizing theoretical models, noise mitigation strategies, and signal conversion techniques. Section “Methodology” outlines the methodological framework employed in this study. The findings are reported in section “Results”, followed by a detailed analysis in section “Analysis”, which examines aspects such as signal generation, noise impact, digital processing, and synchronization precision. Finally, section “Discussion” highlights the outcomes, discusses implications, and suggests avenues for future research, while section “Conclusion” encapsulates the key findings and proposes directions for further exploration.

## Literature review

Quantum synchronization (QS) is a phenomenon that arises from the nonlinear interactions among quantum oscillators. This area of research has gained significant attention due to its promising applications in advanced communication systems and quantum networks. Lohe (2010)^20^ pioneered the concept of phase synchronization in quantum systems, effectively challenging the prevailing belief that synchronization in quantum oscillators with constant linear interactions is unattainable. This work marked a significant advance in the theoretical foundations of QS. Subsequent research has explored the dynamics of dissipative quantum systems, emphasizing the critical role of nonlinear dissipation in achieving robust QS. Koppenhöfer et al. (2019)^21^ experimentally demonstrated QS through digital quantum simulation, representing a vital step toward the practical realization of QS in technological frameworks.

While existing research has leveraged entanglement through time-correlated detection events for synchronization, these studies typically regard quantum clock synchronization as a form of QS. However, our approach diverges fundamentally by exploiting quantum particles’ inherent nonlinear and dissipative properties. This distinction highlights the unique dynamics underpinning our investigation. Quantum clock synchronization using energy-time entangled photons is a related but distinct approach that achieves high precision in time synchronization. Researchers have demonstrated synchronization with remarkable stability and accuracy by harnessing the temporal correlations of entangled photons. These methods enable synchronization at the picosecond level, achieving up to less than $<50$ picoseconds, which is crucial for quantum communication networks that require precise timing^17^. Furthermore, a proposed quantum network integrating satellite- and ground-based clocks through quantum clock synchronization (QCS) aims to achieve picosecond-level accuracy. This innovation enhances secure time synchronization for classical applications and addresses the stringent demands of future quantum networks^22^.

Optical lattice clocks significantly advance precision timekeeping by utilizing quantum transitions in atoms such as strontium and thulium. Unlike cesium atomic clocks, which are affected by Doppler shifts and have broader linewidths, optical lattice clocks provide exceptional stability and precision, with Allan deviations as low as $10^{-18}$^23^. These clocks confine thousands of atoms in a periodic optical potential, achieving femtosecond-level precision, which is crucial for future networks and quantum systems^24^. The output from these clocks is often processed into frequency combs, allowing for integration with both classical and quantum networks. Frequency combs translate the high-frequency outputs of optical lattice clocks into evenly spaced spectral lines suitable for electronic processing. Their coherence across optical and microwave domains is ensured through stabilization techniques like carrier-envelope offset (CEO) correction and phase-locked loops (PLLs)^18^. Frequency combs have been used in satellite-based synchronization^25^. This makes them indispensable for applications such as advanced communication systems and quantum networks. By exploiting controlled dissipation, QNS stabilizes atomic states and achieves synchronization across network nodes^7^. It uses QNS within a dissipative framework to demonstrate QS. This approach achieves exceptional stability at the $10^{-15}$ level^7^, representing a significant advancement in synchronization precision. Further, using frequent combs can be extended for more considerable distances^26^. Moreover, hardware enhancements like mode-locked lasers and ADCs provide cost-effective synchronization for femtosecond timing accuracy in quantum communication. Integrating quantum systems in communication networks could be transformative, but overcoming noise, environmental sensitivity, and integration challenges is essential for advancing beyond 5G/6G.

### Quantum synchronization over the network

Understanding the role of QNS in the present network infrastructure requires a solid mathematical groundwork in synchronization in classical and quantum systems. In classical terms, synchronization involves adjusting the natural rhythmic oscillations of various oscillators to external disturbances, such as electromagnetic fields or other coupling mechanisms. This occurs commonly in complex systems like vacuum-tube radio generators, pendulum clocks, and light-pulsing fireflies. These systems naturally oscillate due to their physical properties and the sustaining energy sources. These oscillators are categorized as self-sustaining or self-oscillatory systems in physics and nonlinear dynamics. Confirming the self-sustaining nature of an oscillator often entails removing it from its environment.

The nonlinear nature of the atom-field interaction and the dissipation of the resonators enable a coherent oscillation frequency to be established across multiple network nodes, potentially surpassing the limitations of classical synchronization methods^27^. For instance, classical synchronization methods like the PTP and NTP face challenges such as network delays, asymmetry, and security vulnerabilities^7^. QS has emerged as a crucial phenomenon in quantum nonlinear dynamics, with potential applications in quantum information processing and communication networks^28^. Research on quantum-enhanced time synchronization indicates that aligning the oscillation frequencies of qubits across network nodes can act as a master clock, enhancing the accuracy and stability of time synchronization^7^. Additionally, a plug-and-play synchronization scheme using mature classical telecommunication and nonlinear optical technologies has been proposed to synchronize quantum network nodes with high precision, reducing the experimental overhead and bypassing the accuracy limitations of current methods^29^.

#### System overview and components

**Fig. 1:**
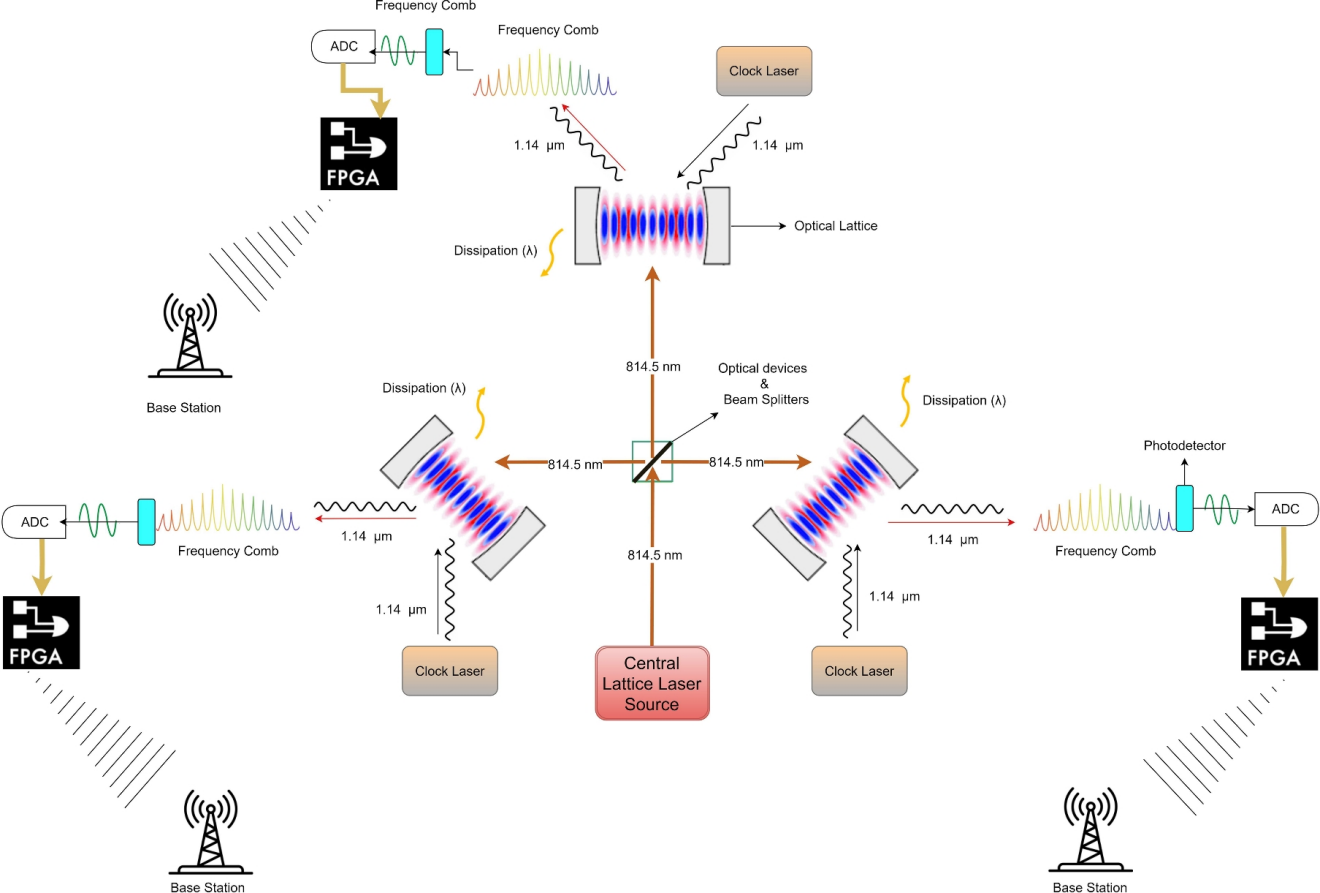
Architecture of quantum nonlinear synchronization (QNS) systems integrated into a communication network. The central lattice laser source generates a coherent light field at 814.5 nm, distributed via optical devices and beam splitters to multiple optical lattice clocks. Each clock includes an optical resonator with nonlinear atoms, synchronized by a continuous wave (CW) clock laser frequency-locked using Pound–Drever–Hall (PDH) locking. A mode-locked laser converts this clock frequency into femtosecond pulses, producing a frequency comb. High-sensitivity photodetectors convert the optical signals from the frequency comb into electrical signals. These analog signals are digitized using high-resolution, high-speed analog-to-digital converters (ADCs), preserving timing accuracy. The digitized signals are processed by field programmable gate arrays (FPGAs) for real-time synchronization, error correction, and data integration. The synchronized signals are transmitted to base stations, ensuring precise timing across the network. Controlled dissipation mechanisms remove excess energy and mitigate decoherence to stabilize the system.

The architecture depicted in Fig. 1 shows a network of three optical lattice clocks connected to a central laser source and exhibiting QNS in the presence of dissipation. The following details the key components and their roles in achieving quantum synchronization.

- *Laser Source* The laser source at the center emits a steady light beam at 814.5 nm, recognized for its high spectral purity and constancy. This continuous beam is sent to the three optical lattice clocks (nodes) through a system of precisely positioned optical fibers, a series of beam splitters (represented by only one beam splitter in the Fig. 1), ensuring minimal phase noise and dispersion^6^.
- *Optical Lattice Clocks (Nodes)* The resonator within each node contains nonlinear atoms, such as thulium, which are confined within a periodic potential created by the interference of multiple laser beams. These resonators are carefully calibrated to ensure that the nonlinear atoms experience a uniform external field, effectively stabilizing their spatial positions and synchronizing their oscillations with remarkable accuracy.
- *Photonic Pumping and Dissipation* Photonic pumping includes introducing a specific number of photons into the resonators using high-power, narrow-linewidth lasers to keep the limit cycle state. To eliminate excess energy, stabilize the synchronized atomic state, and reduce decoherence effects, controlled dissipation methods like precise thermal control and active feedback systems are utilized^7^.
- *Clock Laser* Each node includes a Continuous Wave (CW) clock laser, which is frequency-locked to a particular atomic transition in nonlinear atoms using advanced methods like Pound–Drever–Hall (PDH) locking. This ensures that the clock laser maintains an extremely narrow linewidth and exceptional long-term stability^30^.
- *Frequency Comb and Pulse Train* A stable clock frequency is transformed into a sequence of very short femtosecond pulses by a mode-locked laser. These pulses undergo spectral broadening, creating a frequency comb with evenly spaced spectral lines. This results in the translation of precise timing information into the frequency domain. Nonlinear optical processes drive this process, such as supercontinuum generation in microstructured fibers^31,32^.
- *Photodetectors* Highly sensitive photodetectors transform optical signals from the frequency comb into electric signals. These photodetectors function with minimal noise and wide bandwidth to guarantee precise detection of the intensity of the spectral lines, providing accurate timing information^30^.
- *Analog-to-Digital Conversion (ADC)* The analog electrical signals from the photodetectors are transformed into digital signals by high-resolution and high-speed ADCs. These ADCs provide precise granularity and low jitter to maintain timing accuracy^33^.
- *Field Programmable Gate Array (FPGA)* The signals that have been digitized are processed using FPGA technology. FPGAs offer flexible and high-speed processing capabilities, enabling the real-time execution of intricate algorithms for synchronization, error correction, and data integration. This processing ensures robust and accurate time distribution throughout the communication network^34^.
- *Communication Network Integration* The communication network transmits the processed synchronization signals to base stations. Various devices and subsystems receive the synchronized timing information from the base stations, ensuring coherent operation and precise timekeeping across the entire network.

#### Theoretical framework

Our system’s theoretical foundation is based on self-sustaining oscillators, qubits, phase oscillation, and limit cycles. A self-sustaining system can operate independently without external support, which is essential for the stability and resilience of quantum systems^35^. In quantum computing, a qubit (or quantum bit) is the fundamental unit of quantum information. Unlike classical bits, which can only be in one of two states (0 or 1), a qubit can exist simultaneously in a superposition of both states. This property allows qubits to perform complex computations more efficiently than classical bits. A qubit is typically represented as a two-level quantum system with basis states $|0\rangle$ and $|1\rangle$.

**Fig. 2:**
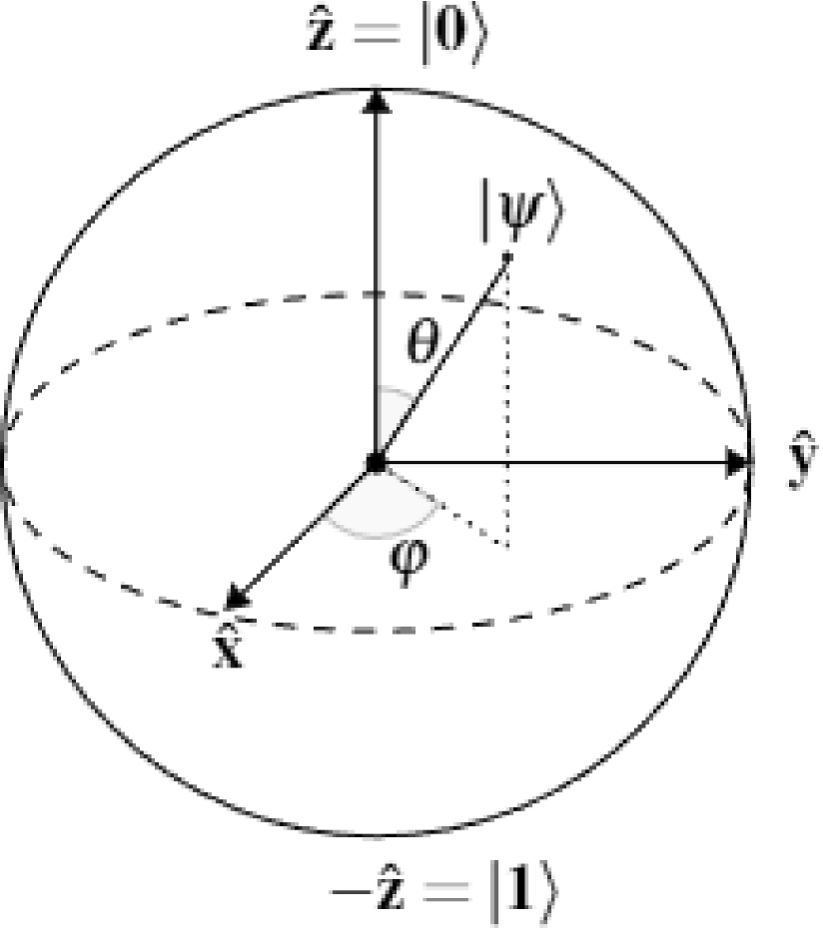
Bloch sphere.

Phase oscillation in quantum systems refers to the periodic variation in the phase of a quantum state. For qubits, this oscillation can be represented on the Bloch Sphere (Fig. 2), where the quantum state is depicted as a point on the sphere’s surface. The phase evolution, governed by the system’s Hamiltonian, causes the state vector to rotate around the sphere over time. Quantum synchronization requires qubits to oscillate at nearly identical frequencies, minimizing phase differences and ensuring coherence. Self-sustaining qubits, which maintain stable and independent oscillation frequencies, are pivotal in building robust quantum systems with enhanced synchronization. A key concept in the study of self-sustaining oscillators is the *limit cycle*, a closed trajectory in phase space defining the oscillator’s stable, periodic behavior. In quantum systems, the evolution of self-sustained oscillators toward their limit cycle dynamics ensures stability even in the presence of noise and disturbances. Limit cycle dynamics provide a robust framework for addressing synchronization challenges, enabling precise control and coordination in quantum networks. The interplay between phase oscillation and limit cycle dynamics forms the foundation for scalable quantum systems, supporting applications in distributed quantum computing, clock synchronization, and quantum-enhanced metrology. These principles enable the development of resilient quantum architectures capable of advanced synchronization and performance in complex environments.

The system’s theoretical framework is based on Rabi oscillations and the Tavis–Cummings Hamiltonian. These illustrate the complex interaction between qubits (atoms) and the external optical field, providing a foundation for understanding synchronization dynamics. The Hamiltonian for the three-qubit system is as follows:

1 $$\begin{aligned} \hat{H} = \hbar \omega _0 \left( \hat{n} + \frac{1}{2}\right) + \hbar \Omega _1 \frac{\hat{\sigma }_z^{(1)}}{2} + \hbar \Omega _2 \frac{\hat{\sigma }_z^{(2)}}{2} + \hbar \Omega _3 \frac{\hat{\sigma }_z^{(3)}}{2} + g \hbar \omega _0 \left( \hat{\sigma }_x^{(1)} + \hat{\sigma }_x^{(2)} + \hat{\sigma }_x^{(3)}\right) \left( \hat{a} + \hat{a}^{\dagger }\right) + f \cos (\omega t)\left( \hat{a} + \hat{a}^{\dagger }\right) \end{aligned}$$

where $\omega _0$ is the natural frequency of the oscillator, $\hat{n}$ is the number operator for the photons in the mode, $\Omega _1, \Omega _2, \Omega _3$ are the Rabi frequencies for the three qubits, $\hat{\sigma }_z^{(i)}$ and $\hat{\sigma }_x^{(i)}$ are the Pauli operators for the *i*-th qubit, *g* is the coupling strength between qubits and photons, *f* is the amplitude of the driving force (defined as $f = \hbar \lambda \sqrt{n_p}$, where $\lambda$ is the coupling coefficient and $n_p$ is the number of photons), and $\hat{a}$ and $\hat{a}^{\dagger }$ are the annihilation and creation operators for the photon mode. A one-drive coupling mechanism synchronizes Individual qubit phases with the external light field. This synchronization is essential for preserving coherence throughout the system. The temporal evolution of the system’s state is described by the density matrix denoted as $\hat{\rho }$. The master equation governs the evolution of this system:

2 $$\begin{aligned} \frac{d}{dt}\hat{\rho } = \frac{1}{i \hbar }[\hat{H}, \hat{\rho }] + \frac{\lambda }{2}(2 \hat{a} \hat{\rho } \hat{a}^{\dagger } - \hat{a}^{\dagger } \hat{a} \hat{\rho } - \hat{\rho } \hat{a} \hat{a}^{\dagger }) \end{aligned}$$

where:

- $\frac{1}{i \hbar }[\hat{H}, \hat{\rho }]$ - term representing the unitary evolution under the Hamiltonian $\hat{H}$
- $\frac{\lambda }{2}(2 \hat{a} \hat{\rho } \hat{a}^{\dagger } - \hat{a}^{\dagger } \hat{a} \hat{\rho } - \hat{\rho } \hat{a} \hat{a}^{\dagger })$ - dissipative term accounting for energy loss mechanisms, with $\lambda$ being the dissipation rate.

Through the use of non-linear synchronization and regulated dissipation, the system is capable of attaining precise synchronization across numerous optical lattice clocks. This method holds great promise for enhancing timekeeping and synchronization in advanced communication networks, such as upcoming 6G technologies. Incorporating mode-locked laser methods and atomic clock lasers also boosts the accuracy and steadiness of the synchronized state, laying the groundwork for progress in quantum technologies.

##### Spectral density

**Fig. 3:**
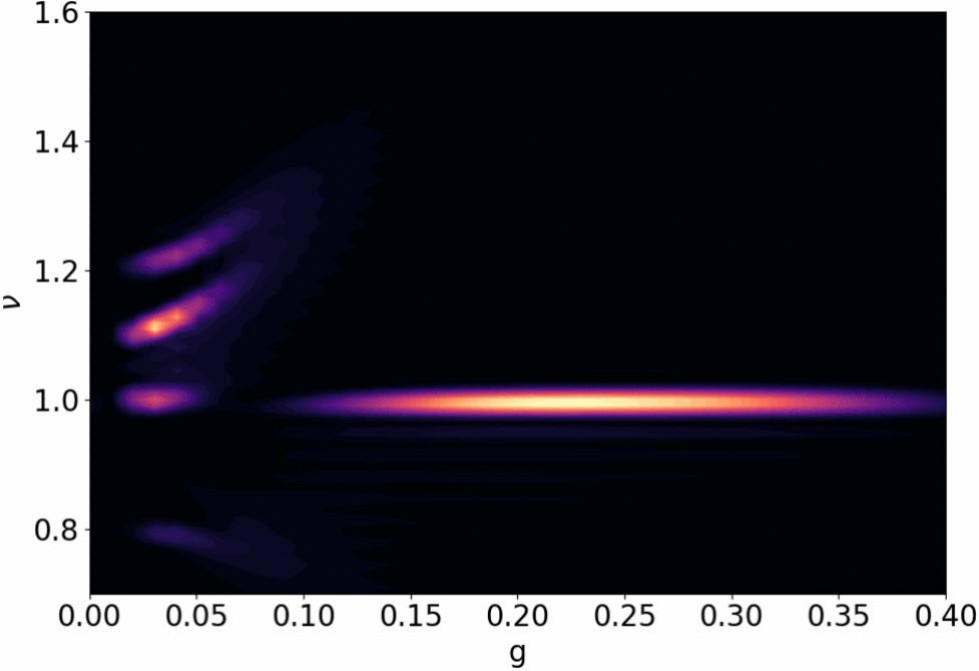
Spectral density plot for three qubits located at different nodes and synchronised with an external optical field ($\omega _0 = 1.0$, $\Omega _1 = 1.1$, $\Omega _2 = 1.2$, $\Omega _3 = 0.8$). As the coupling strength between atom and field increases, the synchronization dominates at the external field frequency.

The spectral density $S(\nu )$^7^of laser-driven qubits can be used to analyze the system synchronization characteristics as shown in Fig. 3. It is the observable intensity of the light inside the resonator with frequency $\nu$ for a fixed coupling *g* and dissipation rate $\lambda$. Solving the master equation at each time step yields the system’s state $\rho$. In Fig. 3 Initially, we observe the oscillation frequencies of each qubit and the external field at low coupling strengths; however, as the coupling strength increases, the oscillation frequencies of individual qubits fade, but the oscillation frequency at the external field strength remains and is visible by demonstrating that only one type of oscillation frequency is observable at high coupling strengths. For more details on parametric specifications, the reader can refer to^7^. Spectral density demonstrates how QNS and dissipation can synchronize a tri-node clock network to the maximum precision in thulium atom-based optical lattice clocks, making it crucial for achieving the clock transition at $1.14~\upmu \,{\text{m}}$^6^.

3 $$\begin{aligned} S(\nu )= \left| \int ^{\infty }_{0} dt \exp \{-i\nu t\}Tr\{\hat{\rho }[ \hat{\sigma }_x^{(1)}+\hat{\sigma }_x^{(2)}+\hat{\sigma }_x^{(3)}]/2\}\right| ^2 \end{aligned}$$

### Optical lattice clocks

#### Why using optical lattice clocks?

Indeed, as we know, the current 5G generation relies on cesium atomic clocks based on oscillation waves. These cesium atomic clocks provide a precision of around 2 ns, which is highly efficient for the current network. However, Doppler shifts in the propagation time within the network may lead to some uncertainties. The part of Fig. 1 contains the optical lattice clock, which is crucial for achieving high precision in synchronization. Optical lattice clocks may offer a promising solution, providing lower frequency and higher stability. This is mainly because optical lattice clocks utilize numerous atoms simultaneously, and the *mean value* is determined, allowing for faster and more accurate measurement times^18^. Optical lattice clocks are also known as quantum clocks because they measure quantum fluctuations with very narrow transitions within strontium atoms, far narrower than the neural-atom clocks. Quantum fluctuations occur when there is a temporary change in energy within a space. These fluctuations arise due to the Heisenberg uncertainty principle, which states that energy and momentum are inherently random and not precisely determined, denoted as $\Delta E \cdot \Delta t \ge \frac{1}{2} h$.

As we transition into the era of 6G and subsequent network paradigms, a deep understanding of quantum synchronization within optical lattice clocks is becoming increasingly essential. This knowledge is crucial for achieving the ultra-precise and reliable network synchronization necessary to support low-latency communications and the development of the Tactile Internet^36^. From this work context, to analyze our QNS system, we must have an optical lattice framework as detailed in^7^. Quantum synchronization with *optical lattice clocks* offers a paradigm shift in the synchronization of time-keeping devices, which is critical for sophisticated telecommunications infrastructure^7,37,38^.

#### How to generate femtosecond levels from optical lattice clocks?

*Optical lattice clocks* provides an excellent platform for investigating and utilizing quantum synchronization. The idea is to have a device that can trap atoms in an optical lattice form. This trap helps to decrease the Doppler shift, critically affecting clock accuracy and atomic interactions. Well-designed traps can enhance the precision of time measurements^39^. The trapped atoms are measured by their frequency transitions between each energy level. Improvements can be made by using thousands of atoms in the lattice form and measuring them at the end. An indicator to determine the clock frequency is determined by the Allan deviation as follows:

4 $$\begin{aligned} \sigma _y(\tau ) \approx \frac{\Delta u}{(v_0 \sqrt{N} \tau )} \end{aligned}$$

where *N* is the total number of oscillators measured in unit time, $\tau$ is the total measurement time, and $\Delta v$ is the variance of the transition frequency $v_0$. To improve this, the idea is to increase $v_0$, which may lead to a minor Allan deviation $\sigma _y (\tau )$. In addition, the trapping of the atoms helps decrease the Allan deviation by extending the coherence interaction time $\Delta t$ between atoms, which leads to minor variance frequency $\Delta v \approx \frac{1}{\delta t}$. The quality factor denoted as $Q = \frac{v_0}{\Delta v}$, of the optical lattice clock, is expected to be higher than that of the microwave clock^40^. In this case, the stability of the optical lattice clock can be achieved $\sigma _y (\tau ) \le 1 \times 10^{-15} \tau ^{-1/2}$. A mode-locked laser can produce pulses within the femtosecond range to achieve femtosecond-level precision for the clock network. Thus, high-frequency stability can be achieved, which can be connected to the 5G network^39^.

Mathematically speaking, once the optical lattice clock generates the pulse, we can measure the Allan deviation using the following equation^41^:

5 $$\begin{aligned} \sigma _{y} (\tau ) = \sqrt{\frac{1}{2M} \sum _{i=0}^{M-1} (y(i+1) - y(i))^2} \end{aligned}$$

where *y* represents the normalized frequency deviation (fractional frequency) over the *i*-th averaging period of duration $\tau$, where $y = \frac{v_i - v_0}{v_0}$. Here, $v_i$ is the measured frequency at the *i*-th time. The *M* represents the total observations (Fig. 2).

#### Pulse generation with optical lattice clocks

To generate pulses from an optical lattice clock, researchers begin with the highly stable continuous wave output from the local clock laser, which is precisely locked to the atomic transition frequency of thulium atoms. This ensures a narrow linewidth and high stability, which is crucial for accurate frequency measurement. The next step involves converting this continuous wave light into ultra-short pulses using a mode-locked laser technique.

The continuous wave light from the clock laser is directed into a mode-locked laser cavity. A gain medium, such as titanium-doped sapphire, is used to amplify the light inside this cavity due to its broad gain bandwidth, which is suitable for producing femtosecond pulses. A mode-locking element, such as a saturable absorber or a Kerr lens, is incorporated into the cavity to achieve mode-locking. The saturable absorber has an intensity-dependent transmission, where higher-intensity light experiences less absorption, promoting the formation of pulses. Alternatively, the Kerr lens relies on intensity-dependent changes in the refractive index to favor pulse formation through self-focusing effects. This mode-locking mechanism forces the laser to emit light as short pulses rather than continuous waves. The optical cavity, consisting of mirrors and the gain medium, forms a resonant structure where the light circulates. The cavity length determines the interval between successive pulses, also known as the repetition rate ($f_r$). The equation for the repetition rate is $f_r = \frac{c}{2L}$, where $c$ represents the speed of light and $L$ represents the cavity length. This results in a series of femtosecond pulses with a consistent repetition rate, typically ranging from hundreds of MHz to a few GHz. This allows for precise time measurement and supports various applications in fundamental research, metrology, and high-precision measurement^30^.

We modeled our pulse to be $E(t)$^42^, mathematically as:

6 $$\begin{aligned} E(t) = E_0 e^{-\frac{1}{2}\left( \frac{t}{\tau }\right) ^2} \cos (2\pi f_0 t + \phi ), \end{aligned}$$

where $E_0$ is the peak electric field amplitude, $\tau$ is the pulse width, $f_0$ is the central frequency of the pulse, and $\phi$ is the phase offset. The time-domain representation of the Gaussian pulse ensures that the bandwidth $\Delta f$ of the pulse is inherently linked to the time-domain width $\tau$ by the time-bandwidth product, a fundamental parameter given by^43^ $\Delta f \cdot \tau \ge \frac{1}{4\pi }$.

To ensure ultimate precision, the optical lattice clocks used in this technique are calibrated to primary cesium standards and are routinely confirmed by international timekeeping bodies^44^. The accuracy of the optical lattice clocks is such that the fractional frequency uncertainty can reach $10^{-18}$, making them ideal candidates for our synchronization system^45^. We must downconvert the high-frequency optical signal to a lower frequency that the communication device can process.

### Frequency combs

Frequency combs are generated through a multi-step process involving ultrashort pulse generation and spectral broadening. Initially, a mode-locked laser produces a train of femtosecond pulses^18^. These pulses are then directed through a highly nonlinear medium, typically a microstructured fiber or integrated waveguide, to broaden their spectrum^46^. This broadening is primarily achieved through nonlinear optical effects, particularly self-phase modulation. Optical frequency combs generally operate in the near-infrared to visible spectrum (approximately 214 THz to 790 THz), with some systems extending into the ultraviolet. The broadened, octave-spanning spectrum is crucial for generating a stable and precise frequency comb. These combs have diverse applications in spectroscopy, metrology, and optical communications, owing to their ability to provide a set of equally spaced, known frequency references across a wide spectral range. Recent advancements include fast-reconfigurable frequency comb systems using electro-optic modulation time-lens techniques and highly nonlinear materials like AlGaAsOI waveguides, which offer improved tunability and efficiency^46^.

The broadened spectrum is then subjected to frequency doubling in a nonlinear crystal to measure and stabilize the offset frequency, $f_0$. This process involves self-referencing, where the low-frequency part of the comb spectrum is doubled and beat against the high-frequency part. The beat note detected provides the offset frequency, $f_0$. A phase-lock loop (PLL) is used to lock $f_0$ to a stable radio frequency (RF) reference, ensuring the stability of the frequency comb. Similarly, the repetition rate, $f_r$, is detected using a high-speed photodetector that converts the optical pulses into an RF signal. Another PLL lock $f_r$ to a stable RF source. By stabilizing both $f_r$ and $f_0$, we can link the optical frequency of the thulium clock transition, $\nu _{\text {clock}}$, to the microwave domain using the frequency comb equation $\nu _n = n f_r + f_0$. By carefully selecting the mode number, $n$, that matches $\nu _{\text {clock}}$, a stabilized microwave signal corresponding to $f_r$ or its harmonics can be extracted. This process provides a precise microwave reference, combining the advanced capabilities of optical frequency metrology with the practical utility of microwave frequency standards, thus offering high precision and stability in frequency measurement.

In recent work, the frequency comb has been generated using a monolithic ultra-high-Q microresonator leveraging Kerr nonlinearity. Unlike traditional mode-locked laser-based combs, Kerr frequency combs utilize the interaction of a continuous-wave pump laser with the resonator modes to produce a broad and stable comb. The intrinsically broadband nature of parametric gain facilitates the generation of discrete comb modes over a $\sim 500 \, \text {nm}$ span (approximately $70 \, \text {THz}$) around $1550 \, \text {nm}$, without external spectral broadening. Measurements based on optical heterodyning reveal that cascaded parametric interactions in the microresonator overcome passive cavity dispersion, resulting in an optical frequency comb with uniform spacing. The mode spacing has been verified with remarkable experimental precision of $7.3 \times 10^{-18}$, highlighting its suitability for high-precision synchronization tasks^47^.

The following section outlines the importance of downconversion and additive white Gaussian noise for Frequency combs.

#### Theoretical framework of frequency combs in downconversion

Mode-locked lasers generate frequency combs by generating a coherent series of phase-locked pulses^18^. Each comb line’s frequency, $f_n$, is given by^48^:

7 $$\begin{aligned} f_n = f_{\text {CEO}} + n \cdot f_{\text {rep}}, \end{aligned}$$

where $f_{\text {CEO}}$ represents the carrier-envelope offset (CEO) frequency and $f_{\text {rep}}$ is the repetition rate of the laser. These combs enable high-accuracy downconversion from optical frequencies to microwave or radio frequency domains. The mathematical representation of the electric fields for the frequency comb signal, $E_{\text {signal}}(t)$, and the local oscillator, $E_{\text {LO}}(t)$, is as follows^49^:

8 $$\begin{aligned} E_{\text {signal}}(t) = \sum _{n=-\infty }^{\infty } \delta (t-nT) \exp (i2\pi nf_0t), \quad and \quad E_{\text {LO}}(t) = E_{\text {LO}} \cos (2\pi f_{\text {LO}}t), \end{aligned}$$

where $E_{\text {signal}}(t)$ symbolizes the electric field of the comb’s signal, consisting of impulses at intervals $T$, inverse to the repetition rate $f_0$, and $E_{\text {LO}}(t)$ denotes the field of the local oscillator with frequency $f_{\text {LO}}$. Upon interaction of these fields, the intensity, $I(t)$, of the combined signal is determined by:

9 $$\begin{aligned} I(t) = |E_{\text {signal}}(t) + E_{\text {LO}}(t)|^2 = E^2_{\text {signal}}(t) + E^2_{\text {LO}}(t) + 2E_{\text {signal}}(t)E_{\text {LO}}(t)\cos (\Delta \omega t), \end{aligned}$$

where $\Delta \omega$ denotes the difference in angular frequency between the signal and the LO. This event produces a beat frequency that permits the signal to be downconverted into the electronic realm, denoted as $E_{\text {downconverted}}(t)$. Thus, the downconverted signal is expressed as follows:

10 $$\begin{aligned} E_{\text {downconverted}}(t) = \sqrt{I(t)} \end{aligned}$$

The precision and stability of frequency combs are essential for advanced applications like dense wavelength-division multiplexing (DWDM), quantum key distribution (QKD) networks, and intricate sensing systems. Techniques for dispersion control and phase noise characterization help mitigate issues related to phase noise and environmental variations that impact the comb’s accuracy. After downconverting the optical signal to a lower frequency, we digitalize the electronic signals generated by the frequency comb.

#### Additive white Gaussian noise in frequency combs

In this study, we focus on additive white Gaussian noise (AWGN) as the primary source of signal degradation in frequency combs. AWGN is characterized by a normal distribution with mean $\mu$ and standard deviation $\sigma$, denoted as $\mathcal {N}(\mu , \sigma ^2)$. Its probability density function is expressed as:

11 $$\begin{aligned} p(x) = \frac{1}{\sqrt{2\pi } \sigma } e^{-\frac{(x-\mu )^2}{2\sigma ^2}} \end{aligned}$$

Here, $\mu$ represents the mean, and $\sigma$ denotes the standard deviation of the distribution. For AWGN, we assume $\mu = 0$, indicating a zero-mean random process. When AWGN is introduced to a signal, it results in:

12 $$\begin{aligned} E_\text {downconverted}^w(t) = E_\text {downconverted}(t) + w(t) \end{aligned}$$

where $E_\text {downconverted}(t)$ is the original signal, and *w*(*t*) represents the additive Gaussian noise.

While AWGN effectively models amplitude noise, it does not accurately capture phase noise, which involves random fluctuations in the phase of the signal. Phase noise is typically frequency-dependent and requires more sophisticated modeling techniques, such as stochastic processes or Auto-Regressive Moving Average (ARMA) models, to accurately represent its impact on frequency combs^50^. In our current analysis, we have incorporated only AWGN into our simulations. This approach allows us to assess the impact of amplitude noise on frequency comb performance. However, for a comprehensive understanding of signal degradation in frequency combs, future work should include detailed modeling of phase noise to capture its significant effects on the stability and coherence of the comb lines. By focusing on AWGN, this study provides insights into one aspect of noise affecting frequency combs, serving as a foundation for more comprehensive analyses that include both amplitude and phase noise components.

### Electronic analog to digital signal conversion

Converting an electronic signal to a digital format is a crucial process that requires both theoretical integrity and practical precision. Analog-to-digital converters (ADCs)^51^ play a vital role in preserving the characteristics of the downconverted electronic signal. The functionality of an ADC relies on sampling and quantization. It transforms a continuous-time signal $E_{\text {downconverted}}'(t)$ into a series of digital values $E_{\text {downconverted}}' [n]$, representing the signal amplitude at discrete time intervals. This process can be expressed as follows^52^:

13 $$\begin{aligned} E_{\text {downconverted}}'[n] = Q\left( E_{\text {downconverted}}'(t) \big |_{t=nT_s}\right) \end{aligned}$$

where $Q(\cdot )$ represents the quantization process, $n$ is an integer number that represents the number of sampling, and $T_s$ is the sampling period. The reciprocal of $T_s$ gives us the sampling frequency $f_s$, which is of fundamental importance. Moreover, the ADCs resolution determines the slightest change in signal level that can be identified, which is important^53^. The resolution is typically expressed in bits. For an ADC with a resolution of $b$ bits, the number of quantization levels $N$ is $N=2^b$, and the quantization error is inversely proportional to this number.

The discrete Fourier transform (DFT)^54^ of the digitized signal can then be used to characterize it:

14 $$\begin{aligned} E_{\text {downconverted}}'[k] = \sum _{n=0}^{N-1} E_{\text {downconverted}}'[n] \cdot e^{-\frac{i2\pi k n}{N}} \end{aligned}$$

where $E_{\text {downconverted}}'[k]$ is the DFT coefficient at frequency index $k$, and $N$ is the total number of samples. Choosing an ADC with a suitably high sample rate and resolution is critical in our methodology to effectively capture the spectrum purity and temporal resolution of the electrical signal obtained from the frequency down-conversion process. This stage of our methodology includes the conversion from analog to digital and the careful calibration of the ADCs to meet the stringent requirements of optical clock synchronization, where even minor differences can lead to significant synchronization errors^55^.

### Digital signal processing for synchronization

FPGAs offer high-speed, reconfigurable digital signal processing (DSP) capabilities, making them ideal for achieving subnanosecond-level synchronization in advanced networks. The digital signal from ADCs is processed in the FPGA’s logic gates and memory blocks to ensure precise synchronization^54^.

Once the signal is in the digital domain, it undergoes DSP operations to reduce noise, extract relevant frequencies, and create synchronization correction signals. These steps are crucial for minimizing timing errors and ensuring reliable performance in real-world applications. The primary DSP steps include:

1. *Noise Filtering* To eliminate unwanted noise, the FPGA employs Finite Impulse Response (FIR) filters. These filters are particularly advantageous due to their linear phase response, which ensures consistent filtering without distorting the phase of the signal^52^. The FIR filter output can be expressed as:
  15 $$\begin{aligned} E_{\text {filtered}}[n] = \sum _{k=0}^{N-1} h[k] \cdot E_{\text {downconverted}}[n-k] \end{aligned}$$
  where $E_{\text {filtered}}[n]$ is the filtered signal, $E_{\text {downconverted}}[n]$ is the input signal, $h[k]$ represents the filter coefficients, and $N$ is the filter length. This ensures the preservation of essential frequencies, such as the beat frequency, while suppressing noise outside the desired bandwidth.
2. *Beat Frequency Detection* Detecting the beat frequency is critical for determining the synchronization state. This is achieved using a phase-locked loop (PLL) integrated within the FPGA^56^. The PLL locks onto the phase of the incoming beat frequency, allowing precise tracking of deviations from the desired synchronization target.
3. *Frequency Measurement and Correction Signal Computation* The frequency of the beat signal can be computed using algorithms such as the Discrete Fourier Transform (DFT) or the computationally efficient Goertzel algorithm. The frequency deviation generates a correction signal that adjusts the system’s synchronization. The correction computation is modeled as:
  16 $$\begin{aligned} V_{\text {corr}}(t) = K_{\text {p}} \cdot \Delta f + K_{\text {i}} \int \Delta f(t) \, dt \end{aligned}$$
  where $V_{\text {corr}}(t)$ is the correction signal, $K_{\text {p}}$ and $K_{\text {i}}$ are proportional and integral gains, respectively, and $\Delta f$ is the frequency deviation.
4. *Application of Correction Signal* The correction signal must be applied iteratively to align the system frequency with the target synchronization reference. This feedback loop ensures that timing errors are reduced to sub-nanosecond precision.

This DSP framework combines advanced filtering techniques, precise frequency measurement, and adaptive feedback control to achieve synchronization at an unprecedented level of accuracy. By leveraging the speed and reconfigurability of FPGAs, the system ensures robust synchronization across large-scale networks, enabling critical applications such as quantum communication, 6G networks, and global timekeeping.

## Methodology

**Fig. 4:**
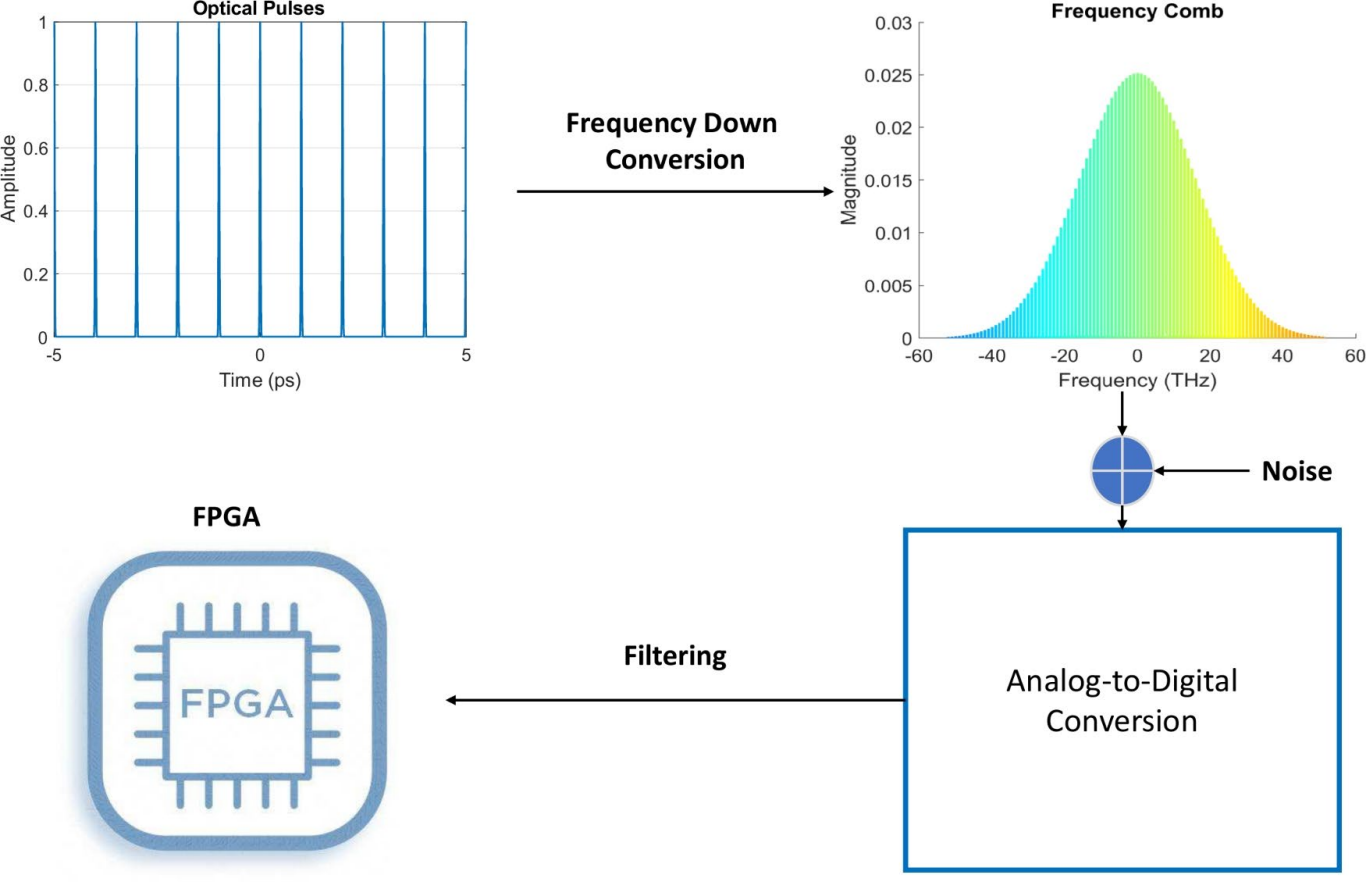
Time synchronization methodology.

The method for achieving optical clock synchronization requires a complex coordination of optical lattice clocks, frequency combs, ADCs, and FPGAs, as illustrated in Fig. 4. This figure builds on the above work (involving one qubit) from Fig. 1, focusing solely on a single optical lattice clock. The process consists of four main steps:

1. Using an *optical lattice framework*, a pulse with ultra-narrow bandwidth and femtosecond precision is generated.
2. A *frequency comb* converts the optical pulse frequency to an electrical signal.
3. The electrical signal is converted from analog to digital.
4. FPGAs-based DSP to generate a clock synchronization correction signal.

### Step 1: pulse generation

An optical lattice clock operates by harnessing the energy levels of atoms to produce an exceptionally accurate time signal. In this system, atoms are confined within a lattice formed by intersecting laser light beams. The frequency of these lasers is carefully tuned to match the natural energy transitions of the trapped atoms. This precise interaction between the atoms and the light establishes the clock’s extraordinary accuracy. The clock achieves femtosecond-level precision (one quadrillionth of a second) by combining several advanced techniques: the controlled manipulation of atomic interactions, ultra-stable lasers to maintain consistent frequencies, and highly sensitive detection systems that measure atomic responses with exceptional accuracy. These features enable the clock to maintain a narrow frequency bandwidth and remarkable stability^15,23^.

### Step 2: frequency down-conversion via frequency comb

Frequency combs generate a sequence of evenly spaced optical frequencies, serving as a precise “ruler” for measuring light in the optical spectrum. These combs are fundamental to applications in spectroscopy, metrology, and telecommunications (Cundiff & Ye, 2003^18^). To measure the optical frequency of a Gaussian pulse, heterodyne detection is used to downconvert it into an electrical signal. This involves pairing the pulse with a local oscillator (LO), typically a continuous wave (CW) laser phase-locked to the frequency comb. When the Gaussian pulse and LO signal overlap, their frequency difference produces an interference pattern. A photodetector captures this interference as a beat signal and converts it into an electrical signal. The frequency of this electrical signal corresponds to the frequency difference between the optical pulse and the LO. This precise mapping allows for accurate measurement of optical frequencies using standard electronics (Udem et al., 2002^13^).

### Step 3: analog-to-digital conversion

Analog-to-digital conversion (ADC) is required for further DSP. ADCs sample electrical signals at a high rate and convert them to digital representations. The resulting digital signal is a discrete approximation of the original electrical signal, with the ADCs’s sampling rate determining its bandwidth.

### Step 4: FPGA-based filters

Field-Programmable Gate Arrays (FPGAs), known for their reprogrammability, are configured to perform specific tasks in our methodology. The FPGA processes the digital signal and generates a corrective signal using the following steps:

1. Noise reduction through digital stream filtering,
2. Detection of the beat signal post-filtering,
3. Precise frequency determination of the beat signal, and
4. Generation of a correction signal for clock synchronization.

This iterative fine-tuning of the correction signal achieves synchronization within femtoseconds.

In our simulation, we focus on the first step, reducing noise by filtering the digital stream. Future work could explore FPGA-based generation of beat signals for communication network devices. This setup is critical for applications such as optical metrology, quantum communication, 6G networks, and navigation. The subsequent sections will discuss the results associated with each step.

## Results

The paper aims to advance femtosecond signal synchronization to picosecond-level devices. To simulate the link between the quantum synchronization stated above, we employ a 263 THz optical signal. It is then downconverted to a lower microwave frequency $\sim$ 100 GHz. This downconverted signal is subjected to white noise and then digitalized. After that, we apply noise filtering using a low pass filter. However, fine-tuning the filtered signal with other steps in FPGAs can be investigated in future studies. The results shown here are based on simulations, and the parameters chosen are based on the investigation of several references. The following are the settings and the results of each particular step. We divide them into five different steps as follows:

1. Signal Generation Optical Lattice Clock
2. Frequency Comb Generation
3. Noise Addition and Impacts
4. Analog-to-Digital Conversion
5. Digital Signal Processing

It is noted that the signal generation process from steps 1 to 2 is performed within a single system. Then, the ADC and DSP (steps 4 and 5) are carried out separately, and it can be practically implemented using an FPGA. Thus, the system from steps 1–2 only considers some noises, i.e., Thermal noises.

### Signal generation using optical lattice clocks

First step of signal generation using optical lattice clocks is fundamental for ultra-precise timekeeping, synchronization, and next-generation quantum networks. Optical lattice clocks operate at optical frequencies, typically ranging from 400 to 700 THz, corresponding to femtosecond ($\textrm{fs}$) time scales. These systems are essential for molecular spectroscopy, high-speed network communication, and global timing networks. In our simulation, we employed a thulium-based optical lattice clock operating at $f = 262.975 \, \text {THz}$, corresponding to a wavelength $\lambda = 1.14 \, \upmu \text {m}$. The time period $T$ of the clock was calculated as:

$$T = \frac{1}{f} = \frac{1}{262.975 \times 10^{12}} \approx 3.802 \, \text {fs}.$$

This setup facilitated the generation of ultrashort pulses with widths below $5 \, \text {fs}$, achieving exceptional timing precision critical for advanced synchronization applications.

**Fig. 5:**
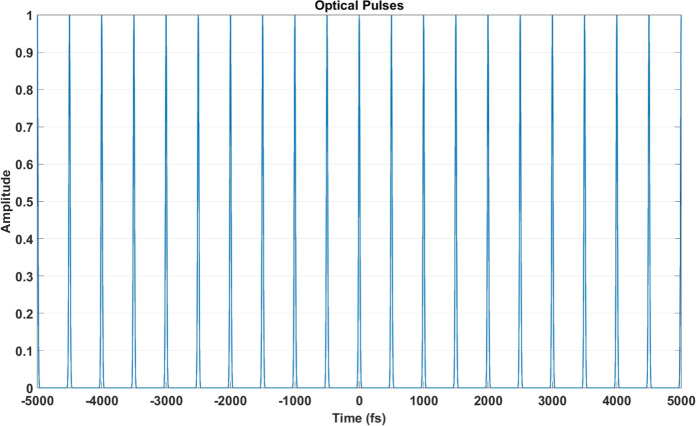
Time-domain representation of optical pulses generated using the thulium optical lattice clock. The pulses exhibit remarkable stability, making them ideal for precision synchronization.

**Fig. 6:**
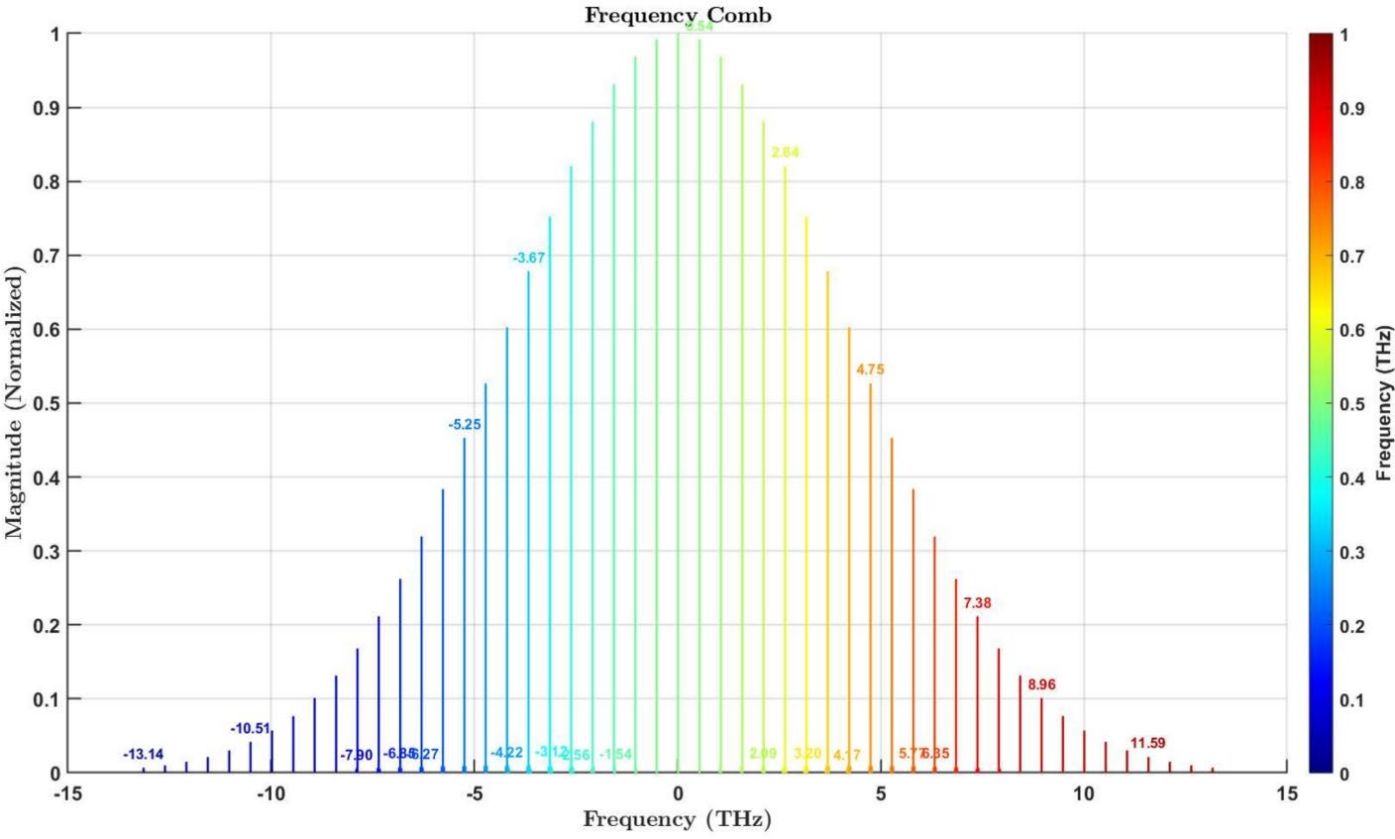
Frequency-domain representation of the optical frequency comb generated using the thulium optical lattice clock. The comb spans a spectral range of $\pm 12.64 \, \text {THz}$ around the carrier frequency, with a repetition rate of $100 \, \text {GHz}$. Each spectral line is equidistantly spaced, normalized to a Gaussian envelope for coherence and minimal dispersion. The amplitude is normalized to 1, highlighting the spectral precision. This frequency comb is instrumental for advanced synchronization tasks, including quantum communication and high-precision metrology.

*Simulation Results:*

- *Time-domain signal* Spanning a temporal range of $[-5, 5] \, \text {ps}$, with a repetition rate of $10 \, \text {fs}$. The periodic and stable femtosecond pulses are depicted in Fig. 5.
- *Frequency-domain signal* A well-defined frequency comb centered at $262.975 \, \text {THz}$, demonstrating stability and precision, as shown in Fig. 6.

### Frequency comb generation

Optical frequency combs (OFCs) have revolutionized precision metrology by providing equidistant frequency markers across broad spectral ranges, linking optical and microwave domains. These systems play a critical role in quantum synchronization, spectroscopy, and high-precision imaging.

**Fig. 7:**
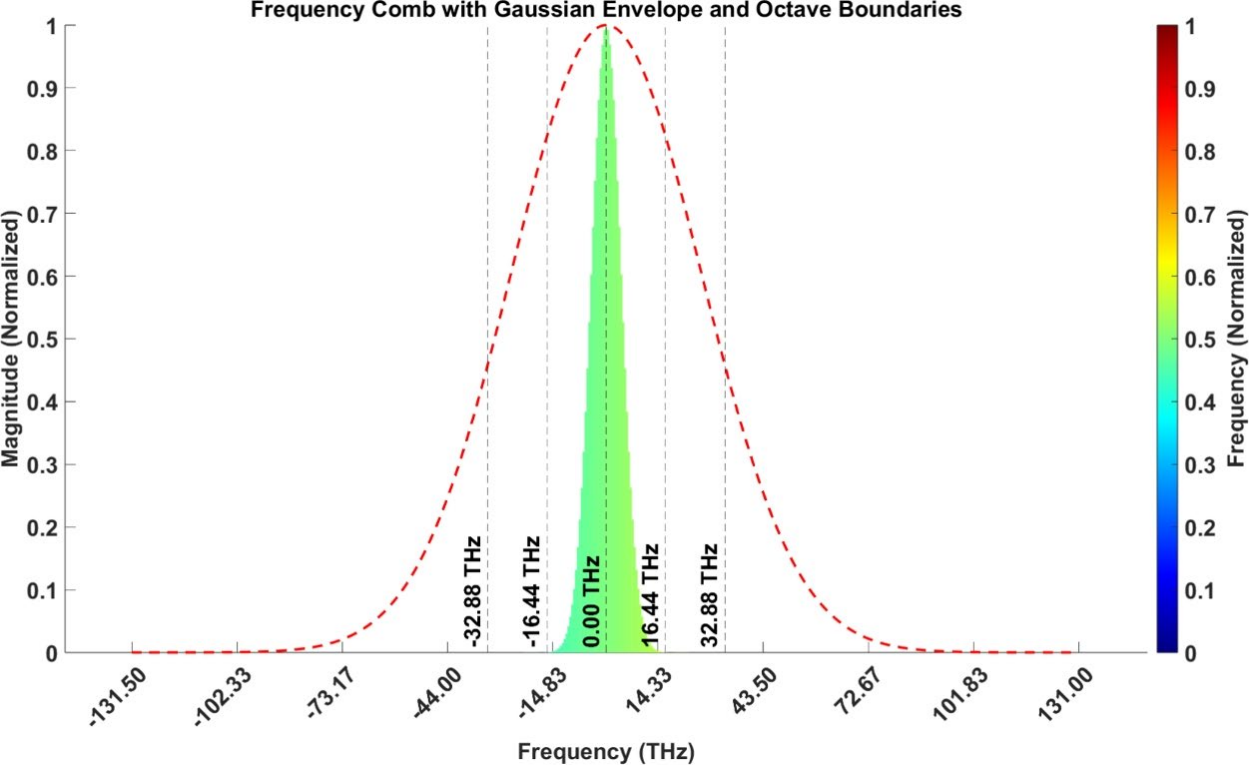
Visualization of the octave-spanning optical frequency comb. The frequency comb lines (green) are symmetrically distributed around the carrier frequency, forming a Gaussian spectral envelope (red dashed curve). The spectral range spans from $131.525 \, \textrm{THz}$ to $394.425 \, \textrm{THz}$, corresponding to a time-domain resolution of $\Delta T \sim 3.802 \, \textrm{fs}$. The octave boundaries (dashed black lines) facilitate $f-2f$ self-referencing, a critical technique for stabilizing the carrier-envelope offset frequency ($f_0$). This stability enables precise synchronization by linking the optical domain to microwave frequencies via downconversion, with a repetition rate ($f_\text {rep}$) of $100 \, \textrm{GHz}$ and a corresponding time interval ($T_\text {rep} = 1 / f_\text {rep}$) of $10 \, \textrm{ps}$. The Gaussian envelope ensures high coherence and minimal dispersion, while the precise spacing of frequency markers supports robust linking of optical and microwave domains. The system has a frequency stability of approximately $\Delta f / f \sim 10^{-15}$. Some advanced systems are achieving even higher stability, nearing $\Delta f / f \sim 10^{-18}$, which allows for synchronization at the femtosecond level in leading-edge communication systems.

*System configuration:* Our frequency comb setup was characterized by the following parameters:

- *Carrier frequency* $$f_c = 262.975 \, \text {THz}$$.
- *Repetition rate* $$f_{\text {rep}} = 100 \, \text {GHz}$$.
- *Total bandwidth* $B = 262.9 \, \text {THz}$, covering a spectral range from $131.525 \, \text {THz}$ to $394.425 \, \text {THz}$, as shown in Fig. 7.
- *Spectral envelope* Gaussian, ensuring high coherence and minimal dispersion across the bandwidth.

*System performance:*

- The frequency comb produces $n = 2630$ equidistant spectral lines symmetrically distributed around $f_c$, ensuring consistent phase coherence.
- The repetition period of the generated comb was calculated as:
  $$T_{\text {rep}} = \frac{1}{f_{\text {rep}}} = 10 \, \text {ps}.$$
- *Octave-spanning comb:* This range allows $f-2f$ self-referencing for precise determination of the carrier-envelope offset frequency ($f_0$), which is crucial for linking optical and microwave domains.

*State-of-the-art comparison:* The performance of our setup aligns with recent breakthroughs:

- **Shin et al.**^57^: Demonstrated terahertz-to-optical synchronization achieving Allan deviations of $10^{-15}$ at 1-second integration.
- **Metcalf et al.**^9^: Developed electro-optic frequency combs spanning $300 \, \text {THz}$, suitable for molecular spectroscopy and quantum communication.

These results highlight the capability of our system to deliver precision and stability comparable to state-of-the-art setups.

### Noise addition and impact

**Fig. 8:**
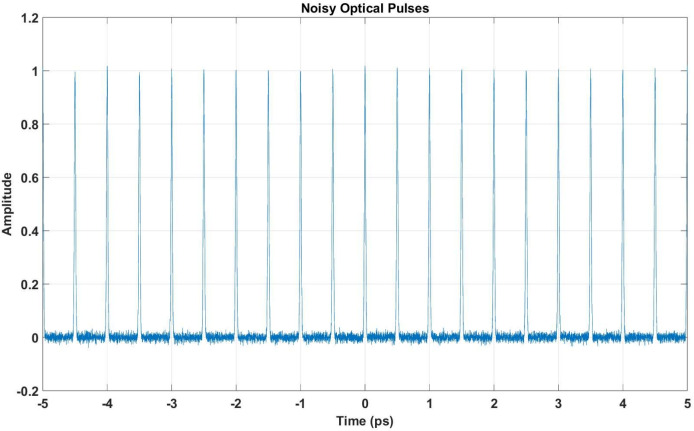
Time-domain representation of the pulse train affected by AWGN. The noise introduces disturbances while retaining the pulse train’s structure.

AWGN is introduced to simulate a real-world signal environment which follows $\mathcal {N} = (0, \sigma ^2)$, where the variation $\sigma ^2 = 0.01$. We choose small $\sigma ^2$ to observe how the performance can handle such a small variation initially.The specific noise figures are important for generating noise at the femtosecond level. When the SNR is low, the noise is also low. In this case, with small steps in synchronization, we may have a low SNR. Thus, the noise figure given is 0.01. The resulting disturbance is observable in the time-domain signal as shown in Fig. 8, where the signal is masked by noise.

### Analog-to-digital conversion

The ADCs are simulated by reducing the sampling rate to $f_s = 100$ GHz or 100 GS/s (gigasamples per second) and limiting the resolution to $b=8$ bits. Consequently, the number of quantization levels is $N = 2^8 = 256$, providing relatively high precision.

**Fig. 9:**
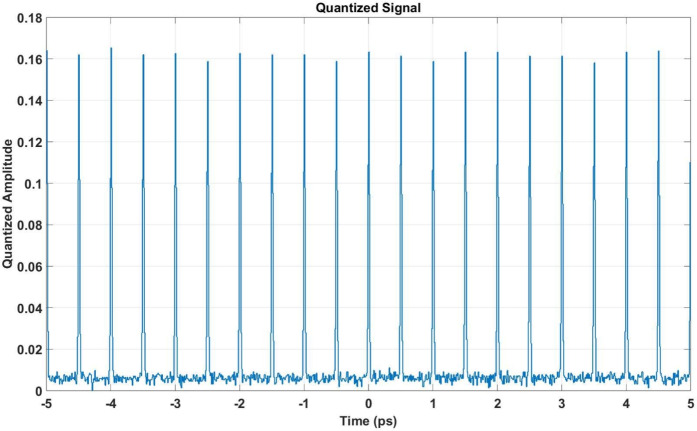
Quantized signal output after ADC. The discretization effect is visible, but the pulse train structure is retained, enabling effective DSP.

The first step involves analyzing the impact of Additive White Gaussian Noise (AWGN) on the pulse train, as shown in Fig. 8. Despite the noise, the overall structure of the pulse train remains intact, allowing further processing. The quantized signal is then generated by ADC, as shown in Fig. 9. The discretization effect is visible in the signal amplitude, but the pulse train’s structure is preserved, enabling effective DSP.

### Digital signal processing

**Fig. 10:**
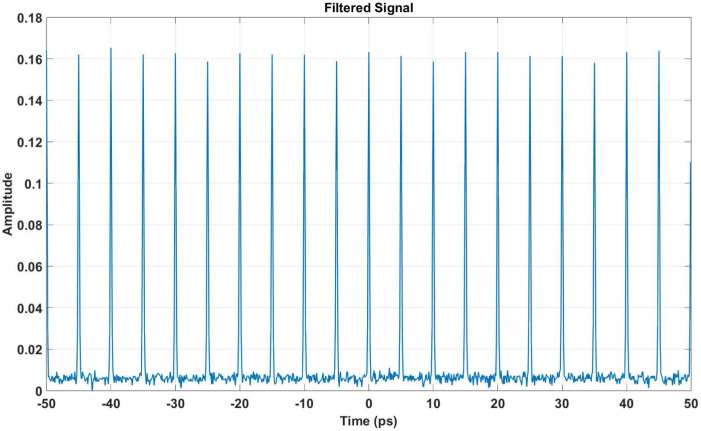
Filtered signal after DSP. Noise components are effectively suppressed, preserving the integrity of the original pulse train.

**Fig. 11:**
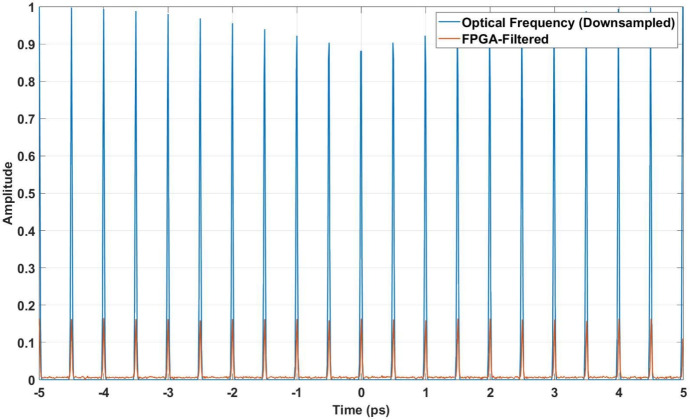
Comparison of the downsampled optical frequency signal with the FPGA-filtered signal. The FPGA-filtered signal demonstrates effective noise mitigation and retains key features of the pulse train.

After the ADCs, a digital filtering process is applied, similar to FPGA-based systems. This filtering reduces high-frequency noise, resulting in a smoother time-domain signal, as illustrated in Fig. 10. The filtered signal displays several peaks, with the interval between each peak measuring 1 ps. This feature is essential for analyzing the formulation and examination of a frequency comb, and it helps assess the impact of noise and DSP techniques on the signal’s key characteristics. A comparison of the downsampled optical frequency signal with the FPGA-filtered signal is shown in Fig. 11. This demonstrates the effectiveness of FPGA filtering in suppressing noise while preserving critical features of the pulse train, ensuring robust synchronization.

### Simulation parameters overview

This section outlines the simulation parameters and evaluation metrics employed to model the synchronization process using optical lattice clocks and frequency combs. These configurations are designed to replicate state-of-the-art synchronization methods as detailed in “sections Signal generation using optical lattice clocks and Frequency comb generation”.

The optical lattice clock operates at a central frequency of $f_c = 262.975 \, \textrm{THz}$, corresponding to a time period of approximately $T_c = 3.802 \, \textrm{fs}$. The Gaussian pulse train is generated with a pulse width of $\tau _{\text {pulse}} = 10 \, \textrm{fs}$ and a repetition rate of $T_{\text {rep}} = 0.5 \, \textrm{ps}$, resulting in 21 periodic pulses spanning a time interval of $[-5, 5] \, \textrm{ps}$. These parameters ensure stable, femtosecond-level timing precision for clock signal generation. Following the optical clock signal generation, a frequency comb is simulated with $n = 2630$ spectral lines centered at $f_c = 262.975 \, \textrm{THz}$. The repetition rate of the frequency comb is $f_{\text {rep}} = 100 \, \textrm{GHz}$, resulting in equidistant spectral lines with a Gaussian envelope to maintain spectral coherence. The simulation incorporates an analog-to-digital conversion (ADC) process, configured with a sampling rate of $f_{\text {ADC}} = 100 \, \mathrm {Sa/S}$ and an 8-bit resolution. To replicate realistic conditions, Gaussian noise (signal-to-noise ratio, $\textrm{SNR} = 40 \, \textrm{dB}$) and phase noise are introduced into the system. Post-processing is performed using FPGA-based digital filters to enhance signal quality. The evaluation metrics include amplitude stability, noise tolerance, and synchronization precision.

#### Evaluation metrics

The following key metrics are computed during the simulation:

- *MaxAmplitudeOriginal* The maximum amplitude of the original pulse train, normalized to $1.0000$.
- *MaxAmplitudeNoisy* The maximum amplitude after Gaussian noise is applied, reflecting real-world conditions ($1.2183$).
- *ADCOutputRange* The amplitude range of the quantized signal after ADC ($0.15553$).
- *FPGAFilteredSignalAmplitude* The amplitude of the filtered signal post-FPGA processing ($0.15553$).
- *Allan Deviation* A measure of frequency stability for the FPGA-filtered signal, calculated relative to the reference frequency ($5.8755 \times 10^{-15}$).
- *Minimum Mean Squared Error (MMSE)* The average squared error between the filtered and original signals ($2.60799 \times 10^{-5}$).

#### Summary of parameters and results

The simulation results and parameter configurations are summarized in Tables 1 and 2.

**Table 1:** Simulation parameters for optical lattice clock and ADC system.

Simulation parameter	Value
Optical Lattice Clock Frequency	$262.975 \, \textrm{THz}$
Time Period of Clock Signal	$3.802 \, \textrm{fs}$
Pulse Width ($\tau _{\text {pulse}}$)	$10 \, \textrm{fs}$
Repetition Rate ($T_{\text {rep}}$)	$0.5 \, \textrm{ps}$
Frequency Comb Repetition Rate ($f_{\text {rep}}$)	$100 \, \textrm{GHz}$
ADC Sampling Rate ($f_{\text {ADC}}$)	$100 \, \mathrm {Sa/s}$
ADC Resolution	8 bits
Signal-to-Noise Ratio (SNR)	$40 \, \textrm{dB}$

**Table 2:** Evaluation results of the synchronization simulation.

Evaluation metric	Obtained value
Max Amplitude (Original Signal)	1.0000
Max Amplitude (Noisy Signal)	1.2183
ADC Output Range	0.15553
FPGA Filtered Signal Amplitude	0.15553
Allan Deviation	$5.8755 \times 10^{-15}$
MMSE	$2.60799 \times 10^{-5}$

## Analysis

### Setup

The synchronization performance of the system was evaluated by comparing the filtered ADC signal to the downconverted optical lattice clock signal. The optical lattice clock operates at $262.975 \, \textrm{THz}$, corresponding to a temporal resolution of $3.802 \, \textrm{fs}$, while the filtered signal operates at $100 \, \textrm{GHz}$. Over the same time interval of $[-5, 5] \, \textrm{ps}$, the optical lattice clock generates $2629.75$ samples, and the filtered signal produces $100$ samples. To ensure comparability, the higher-frequency optical lattice clock signal was aligned to match the resolution of the filtered signal. This adjustment maintained the fidelity of the clock’s timing characteristics for synchronization evaluation.

### Mean squared error (MSE)

The Mean Squared Error (MSE) was calculated to quantify the accuracy of synchronization between the down-converted optical lattice clock signal $E_{\text {downconverted}}'(t)$ and the filtered signal $V_{\text {corr}}(t)$:

$$\text {MSE} = \frac{1}{T} \sum _{t=0}^{T-1} \left| E_{\text {downconverted}}'(t) - V_{\text {corr}}(t) \right| ^2$$

The calculated Minimum Mean Squared Error (MMSE) $2.6 \times 10^{-5}$, which indicates strong agreement between the two signals. Although minor discrepancies in amplitude were observed as a result of energy loss during filtering, the spacing between pulses remained consistent, preserving the synchronization timing. Figure 11 illustrates this consistency in the time domain.

### Cross-correlation analysis

Cross-correlation is a widely used method to evaluate the similarity between two time-series signals by identifying the extent to which one signal aligns with a time-shifted version of the other. In this study, the cross-correlation between the downconverted signal, $E'_\text {downconverted}(t)$, and the FPGA-filtered signal, $V_\text {corr}(t)$, both of size $N=100$, was computed. Each component of the signals is represented as $n=\{1, 2, \cdots , N\}$.

The cross-correlation is mathematically defined as follows^58^:

17 $$\begin{aligned} \hat{R}_{xy}(m) = {\left\{ \begin{array}{ll} \sum \limits _{n=0}^{N-m-1} E'_\text {downconverted}(n+m) V_\text {corr}^{*}(n) & m > 0, \\ \hat{R}_{yx}^{*}(-m) & m < 0, \end{array}\right. } \end{aligned}$$

where *m* denotes the lag displacement, $E'_\text {downconverted}(n+m)$ is the shifted signal, and $V_\text {corr}^{*}(n)$ represents the complex conjugate of the second signal. Positive values of *m* correspond to leading shifts of $E'_\text {downconverted}$ relative to $V_\text {corr}$, while negative values correspond to lagging shifts.

## Cross-correlation versus SNR evaluation

In this section, the impact of SNR on the synchronization performance of the proposed system is analyzed. Two main aspects are evaluated: (1) the cross-correlation performance at $20 \, \textrm{dB}$ and $40 \, \textrm{dB}$ SNR, and (2) the variation of the maximum correlation value as a function of SNR. Further, the system’s performance in low-SNR conditions is thoroughly evaluated, focusing on its resilience to noise.

### Cross-Correlation Analysis

**Fig. 12:**
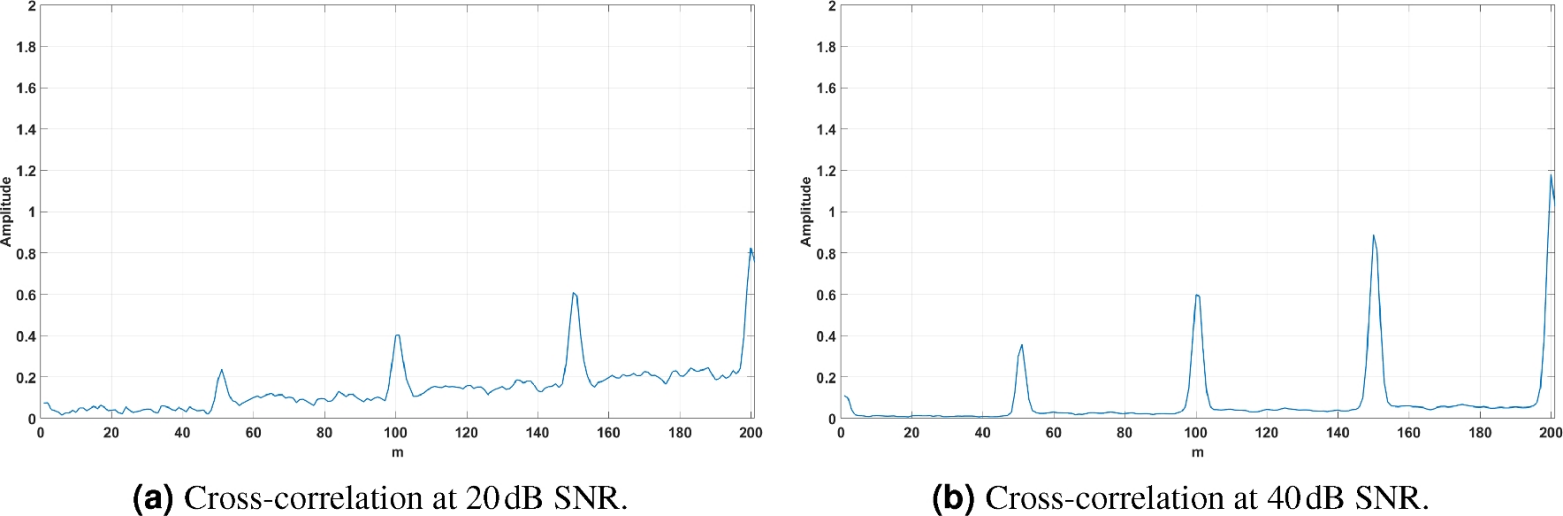
Cross-correlation analysis for signals at $20 \, \textrm{dB}$ and $40 \, \textrm{dB}$ SNR. Peaks correspond to the timing offset between signals.

The cross-correlation analysis for signals at $20 \, \textrm{dB}$ and $40 \, \textrm{dB}$ SNR is shown in Fig. 12. The peaks in the cross-correlation correspond to the timing offset between the signals.

*Observations:*

- At $20 \, \textrm{dB}$ SNR (Fig. 12a), the cross-correlation shows significant noise interference, with less distinct peak values. This highlights the challenge of accurate timing offset estimation in low-SNR environments.
- At $40 \, \textrm{dB}$ SNR (Fig. 12b), the cross-correlation result demonstrates significantly clearer peaks, reflecting improved signal quality and better synchronization accuracy. Noise influence is minimal at this SNR level.

### Variation of maximum correlation with SNR

**Fig. 13:**
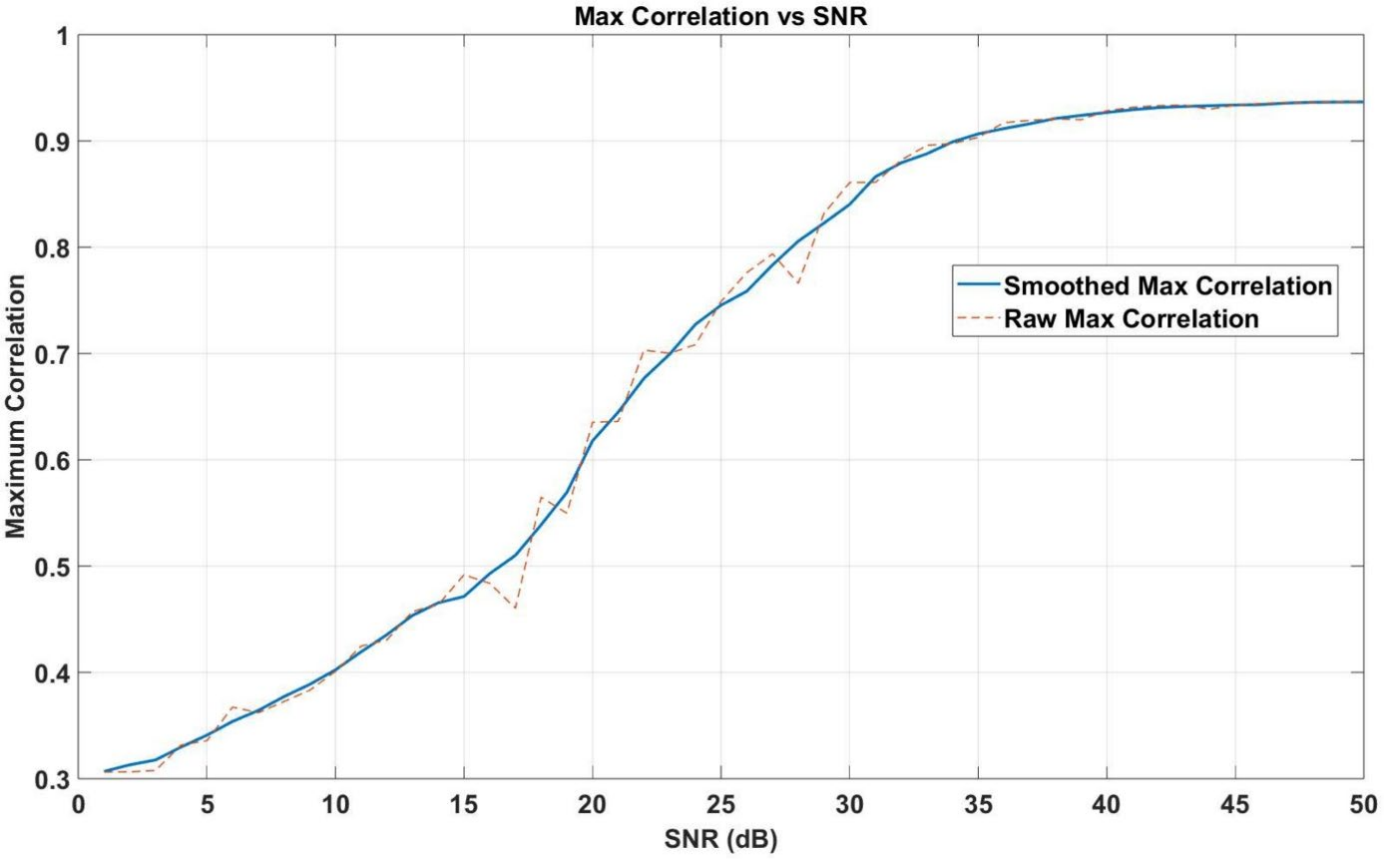
Maximum correlation value as a function of SNR. Both Smoothed Max and Raw Max are shown to highlight the role of noise reduction in improving synchronization accuracy.

Figure 13 shows how the maximum correlation value varies with SNR. The system’s performance can be divided into three distinct regimes:

- *Low SNR Regime (SNR *$<15 \, \textrm{dB}$): In this regime, synchronization performance is significantly degraded due to noise. However, the system retains functionality, as it can still extract useful timing information, albeit with reduced precision. This demonstrates a degree of resilience, which will be further quantified in future comparisons with state-of-the-art protocols.
- *Moderate SNR Transition (SNR *$15-30 \, \textrm{dB}$): As SNR increases, synchronization improves steadily. Maximum correlation values rise progressively, indicating better signal alignment and fewer timing errors.
- *High SNR Stability (SNR *$>30 \, \textrm{dB}$): At higher SNR values, the maximum correlation approaches unity, signifying near-perfect synchronization accuracy. This stability makes the method well-suited for noise-limited environments and advanced communication applications.

#### Smoothed max versus raw max

Figure 13 displays both Smoothed Max and Raw Max correlation values to illustrate the system’s performance. Smoothed Max values are calculated using a moving average, while Raw Max values are more affected by noise fluctuations, highlighting the need for noise reduction algorithms to achieve accurate synchronization^60^.

### Allan deviation

The stability of the synchronization was further evaluated using the Allan deviation. The filtered signal $V_{\text {corr}}(t)$ was transformed into the frequency domain using Fast Fourier Transform (FFT), resulting in $f_{\text {corr}}$, which was normalized concerning the center frequency $v_0 = 262.975 \, \textrm{THz}$. The Allan deviation was calculated as follows:

$$\sigma _y^2(\tau ) = \frac{1}{2 (M-1)} \sum _{k=1}^{M-1} \left( \overline{f}_{k+1} - \overline{f}_k \right) ^2$$

The computed Allan deviation of $5.8755 \times 10^{-15}$ provides exceptional frequency stability, meeting the stringent requirements for femtosecond-level synchronization.

### Implications

The low MSE and high cross-correlation values confirm the effectiveness of the proposed QNS system in achieving reliable synchronization. The Allan deviation indicates the system’s stability, highlighting its suitability for precision timing applications in advanced communication networks, quantum systems, and global timekeeping infrastructures.

## Discussion

The advent of 5G and the forthcoming 6G technologies highlight the indispensable role of URLLC across various domains, including large-scale IoT networks, autonomous vehicles, and smart city infrastructures. Fundamental to URLLC is the necessity for stringent network time synchronization, ensuring real-time and isochronous communication, particularly crucial for applications in beyond 5G systems.

Research highlights the critical role of precise timing in addressing challenges and meeting the rigorous demands of URLLC. This involves disseminating high-resolution temporal data to devices and incorporating mechanisms within the 5G radio interface for wireless synchronization, which is critical to meeting the timing constraints of time-sensitive URLLC applications. Furthermore, with the advent of 6G on the horizon, there is a need for even more precise and reliable synchronization solutions to support the integration of land, air, and maritime communications, enabling ultra-low latency services for a wide range of connected devices.

The precision of synchronization technology has important consequences for quantum communications as well^60^. Quantum communication technologies, such as quantum key distribution (QKD), rely on precisely aligned phase-critical activities to sustain quantum coherence. Timing inconsistencies ignored by standard synchronization methods may result in quantum decoherence, compromising the fidelity and security of quantum communication. Advanced synchronization algorithms that reduce synchronization errors to the femtosecond level significantly reduce the risk of quantum decoherence, increasing the security and dependability of quantum communication networks.

However, despite the transformative potential of advanced synchronization techniques, they present several practical challenges that hinder their widespread adoption. For example, the susceptibility of optical lattice clocks and frequency combs to environmental factors requires robust control mechanisms to ensure consistent performance. Furthermore, integrating these advanced synchronization technologies into existing network software stacks (e.g., the Linux timing subsystems and the jiffies mechanism^61^), which are currently optimized for nanosecond resolution timestamps, presents adaptation challenges.

Addressing these challenges requires collaborative research efforts aimed at improving synchronization resilience to environmental disturbances, expanding its operational scope, and reducing costs through miniaturization and improved efficiency. Furthermore, integration with quantum-resistant cryptographic systems is essential to strengthen communication networks against emerging quantum threats.

In summary, the evolution of communication technologies requires innovative approaches to achieve precise synchronization, which is fundamental for both URLLC and quantum communications. By addressing practical challenges and integrating advanced synchronization techniques with robust encryption methods, communications networks can unlock their full potential in the beyond 5G era, ensuring secure, reliable and low-latency connectivity between different applications and environments.

## Conclusion

This paper introduces a novel approach for achieving femtosecond-level synchronization in optical communication networks by integrating advanced technologies such as optical lattice clocks, frequency combs, ADCs, FPGAs, and QNS. It outlines a framework that combines cutting-edge optical and electronic systems while utilizing innovative QNS applications to meet the growing demand for ultra-precise timing in next-generation networks like 6G and the quantum internet.

QNS employs nonlinear dynamics within atomic-field interactions in an optical resonator, providing a robust and scalable solution for synchronization with unprecedented precision. Every stage in the methodology, from generating stable Gaussian-shaped optical pulses to refining and stabilizing digital signals, demonstrates the significant potential of precise timing to provide transformative changes in communication systems. This work goes beyond optical networks and quantum communication and impacts fields such as deep-space telemetry, navigation, and precision metrology. Future research should focus on improving noise reduction, frequency detection, and ADC performance to achieve attosecond-level synchronization. Advancing quantum noise suppression and integrating it with current methods could lead to robust and precise timing solutions. This study provides a strong foundation for future breakthroughs in optical metrology, quantum communication, and next-generation networks, where precise synchronization will be key.

## Data Availability

The data generated and analyzed during this study are fully included within the article. Reproducibility of the results is ensured through the detailed parameters provided in Table 1, Table 2, and the Results section. Furthermore, the MATLAB code associated with this research is available upon request to facilitate further exploration, address specific inquiries, or support collaborative efforts with academic and research institutions. For further information regarding the data, readers may contact muhammad_idham.habibie@tu-dresden.de.
